# Renal angiomyoadenomatous tumor (RAT): a rare distinct entity with diagnostic challenges—a case report

**DOI:** 10.1186/s43046-020-00056-y

**Published:** 2021-01-07

**Authors:** Ankur Majumder, Ravi Hari Phulware, Arvind Ahuja, Anurag Singla, Pawan Kumar

**Affiliations:** 1 Department of Pathology, ABVIMS, PGIMER, RML Hospital, New Delhi, India; 2 Department of Pathology, All India Institute of Medical Sciences (AIIMS), Room no. C-2, Level 3, Rishikesh, India; 3 Department of Urology and Renal Transplant, PGIMER, ABVIMS, RML Hospital, New Delhi, India; 4 Department of Radiology, Goa Medical College, Bambolim, Goa India

**Keywords:** Rare renal tumor, Renal angiomyoadenomatous tumor, Renal cell carcinoma, Mixed epithelial and stromal tumor, Case report

## Abstract

**Background:**

Renal angiomyoadenomatous tumor (RAT) is a recently described rare renal neoplasm with variations in the presentation, gross, and microscopic findings, and having a benign course and good prognosis. It is characterized microscopically by the admixture of three components—epithelial cells arranged in tubules and nests, angiomyomatous stroma, and capillary-sized interconnecting vascular channels in close association with the epithelial cell clusters. Microscopically, these tumors can be confused with clear cell carcinoma, papillary carcinoma, mixed epithelial and stromal tumors, and angiomyolipoma. RAT differs from conventional clear cell carcinomas, which can rarely be associated with an identical leiomyomatosis stroma occasionally forming abortive vascular structures. RAT is a distinct morphologic entity, being different morphologically, immunohistochemically, and genetically from all renal tumors including conventional clear cell carcinoma and mixed epithelial and stromal tumor of the kidney.

**Case presentation:**

Here, we report a case of a 21-year-old man with renal angiomyoadenomatous tumor, a rare neoplasm with only a few previous cases reported in the literature. Unlike our case, most tumors have been identified in middle-aged males; they present as well-circumscribed, encapsulated tan-brown masses with variably prominent cystic areas.

**Conclusion:**

Diagnosis of RAT is challenging because of the rarity of the disease and common presenting symptoms to other renal pathology and is supplemented with histopathology and immunohistochemistry. A multidisciplinary team approach for diagnosis and management along with long-term follow-up are warranted.

## Background

Renal angiomyoadenomatous tumor (RAT) is a very rare neoplasm with fewer than 15 cases reported in literature till now. Newer entities are being added continuously to the already existing database of tumors and RAT is one such entity that has not yet found its place in the World Health Organization (WHO) classification of kidney tumors. It has been microscopically described as a neoplasm containing an epithelial component in the form of ducts, a stroma that is leiomyomatosis in nature and interspersed by abortive vascular channels [1, 2].

Here, we present a case of RAT in a 21-year-old male with a distinctive presentation and gross features in an attempt to include diversity to the pathological profile of this, particularly rare neoplasm.

## Case presentation

**Fig. 1:**
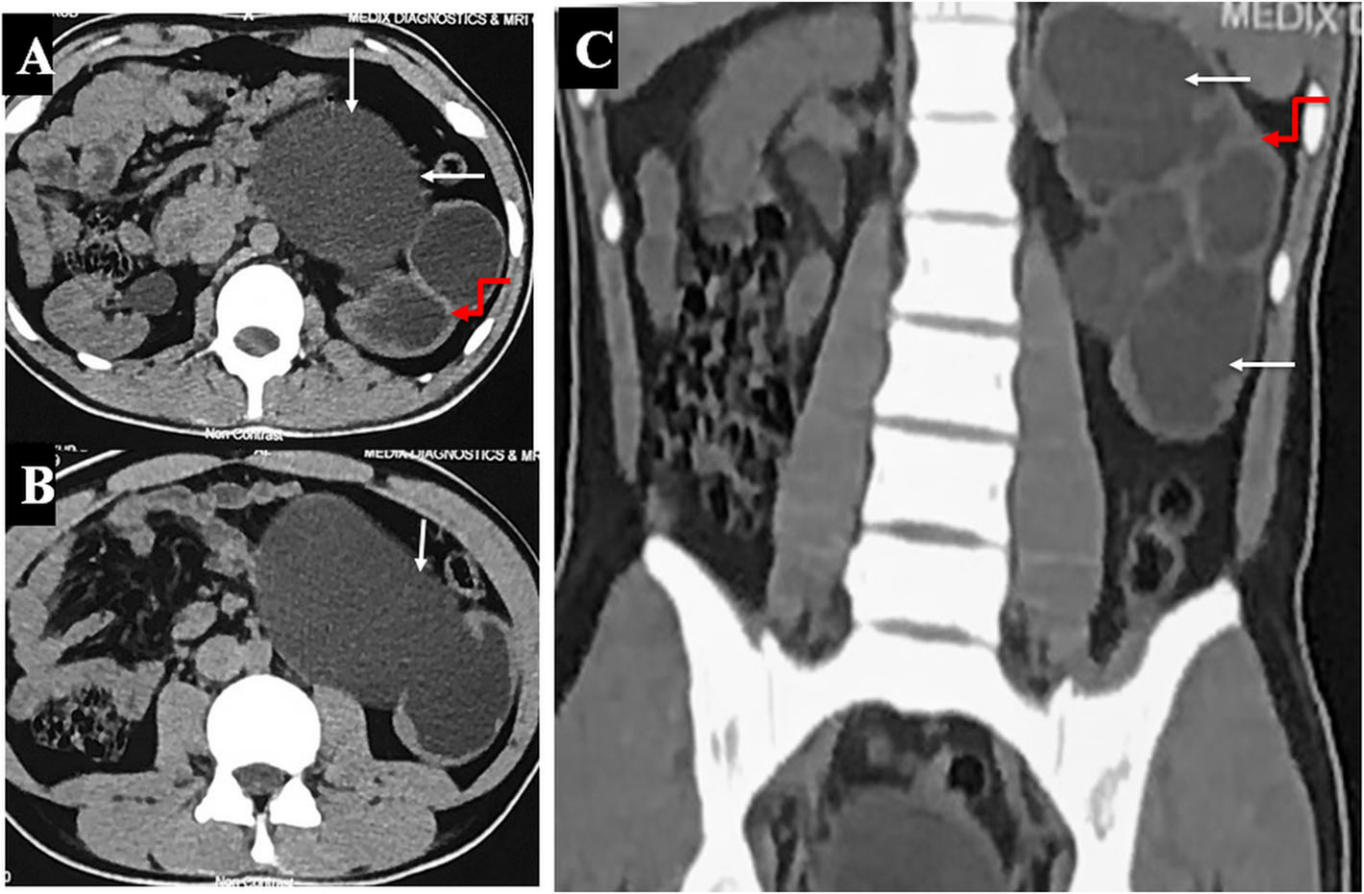
Axial (**a**, **b**) and coronal (**c**) non-contrast-enhanced CT (NCCT) scan images showing gross hydronephrosis of the left kidney (white arrows) with severe parenchymal thinning (curved red arrows)

A 21-year-old male patient presented to the Ram Manohar Lohia (RML) Hospital, Post Graduate Institute of Medical Sciences (PGIMER), New Delhi, India, with pain in left flank pain intermittent hematuria. Non-contrast computed tomography (NCCT) scan of the abdomen showed gross hydronephrosis of the left kidney (white arrows) with severe parenchymal thinning with pelvic ureteric junction obstruction (PUJO) (Fig. 1). A left radical nephrectomy was done with hilar lymph node dissection with a clinical diagnosis of non-functioning kidney secondary to left PUJO.

**Fig. 2:**
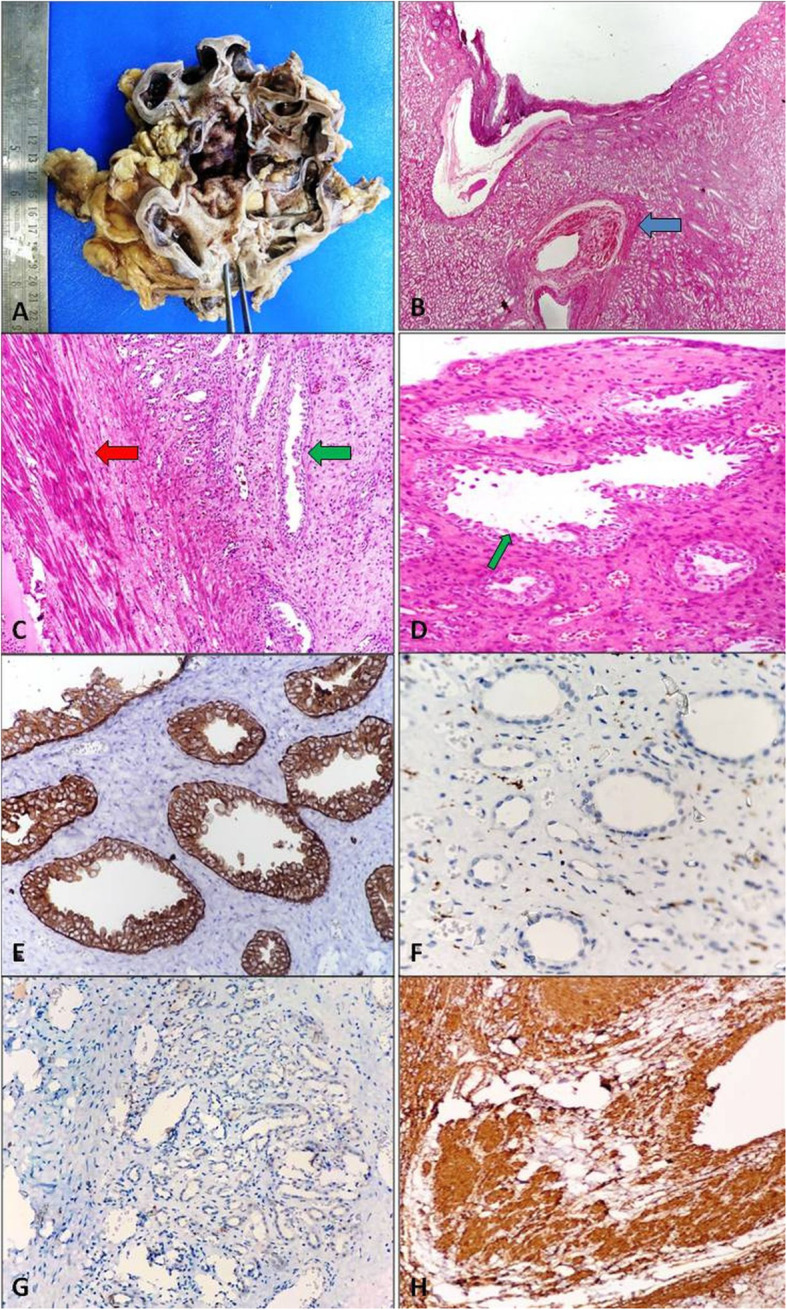
Histopathological examination of renal angiomyoadenomatous tumor (RAT): gross image demonstrating multiple interconnecting cystic spaces with intervening thickened areas with patchy hemorrhagic areas (**a**). Hematoxylin and eosin (H&E) stained lower magnification image showing cystic spaces (epithelial component) along with thick-walled blood vessels (blue arrow) in renal parenchyma (**b**, **h**&**e**, × 40). Epithelial component arranged in adenomatous/glandular/tubular pattern with basally located bland small beaded nuclei and intimately surrounded by thin vascular channels. The cytoplasm of the cells is optically clear with blisters like apical snouts giving the appearance of “Shark’s Smile/moth-eaten” (green arrow). Stromal smooth muscle component (red arrow) (H&E; × 100, **c**; and × 400, **d**). The epithelial component is immunopositive for pan-cytokeratin (**e**, × 400), immunonegative for CD10 (**f**, × 400), and immunonegative for HMB-45 (**g**, × 40). The stromal smooth muscle was immunopositive for smooth muscle actin (SMA) (**h**, × 100)

Gross examination showed a specimen of the kidney measuring 14 × 8 × 6 cm. The external surface was bosselated. The capsule was easily stripped off. No scars were noted externally. Serial slicing revealed multiple interconnecting cysts with few thickened areas replacing the entire parenchyma of the kidney. The cysts ranged in size from 1.5 to 4 cm. Cortico-medullary differentiation could not be made out (Fig. 2a). A small part of the ureter was seen measuring 1 cm. Hilar lymph nodes measuring 2.5 × 1 was received separately.

Microscopic examinations from multiple sections showed a tumor composed of epithelial, smooth muscle and vascular components. The epithelial component was arranged in the form of tubules. The tumor cells had a moderate amount of clear to eosinophilic cytoplasm with a basally placed round to oval vesicular nucleus and apical snouting. Intervening stromal areas showed smooth muscle differentiation arranged in fascicles and bundles (Fig. 2c, d). For the vascular component, both thick and thin blood vessels were seen which were lined by plump endothelial cells. The resected end of the ureter and the hilar structures were free of the tumor. The specimen labeled as hilar lymph node showed features of reactive lymphoid hyperplasia. Immunohistochemistry (IHC) was done for the tumor. The epithelial component was positive for pan-cytokeratins (Fig. 2e) and was negative for CD10 (Fig. 2f) and HMB-45 (Fig. 2g), the stromal component was positive for smooth muscle actin (SMA) (Fig. 2h) and was immunonegative for CD34, estrogen receptor (ER); thereby confirming the smooth muscle differentiation. Therefore, based on the histomorphological and immunohistochemical staining pattern diagnosis of renal angiomyoadenomatous tumor was given.

## Discussion

RAT is a rare and distinct neoplasm. The average age group in reported cases was 46 years. No sex predilection has been noted in the tumor. Rare studies have shown a tumor with cystic changes. Common differentials for mixed renal carcinomas to be kept in mind are mixed epithelial and stromal tumor of the kidney (MESTK), angiomyolipomas, clear cell renal cell carcinomas with angioleiomyomatous stroma, and clear cell papillary renal cell carcinomas (ccpRCC) [3–5].

According to Michal et al. [2], RAT epithelial component consists of adenomatous structures composed of cells that are secretory having basophilic nuclei alienated along the basal membrane and prominent apical snouts, resulting in a characteristic appearance of “Shark’s smile.” This epithelial component is usually shown immunopositivity for all cytokeratins like CK-7 more than CK-20, CAM 5.2, and cytokeratins AE1-AE3. In addition to cytokeratins positivity, epithelial membrane antigen (EMA) and vimentin are also positive. While IHC for CD10, Melan-A, and HMB-45 is immunonegative. The present case showed immunopositivity for pan-cytokeratin, while IHC foe CD10 and HMB-45 were negative. Focal solid and clear cell areas may be seen. It may resemble conventional clear cell carcinoma Fuhrman grade 1. These secretory cells usually contain glycogen which is periodic acid-Schiff (PAS) positive, diastase resistant, and mucicarmine negative [2–4]. Our case shows similar morphology.

RAT shows a unique relation between the capillary network and the epithelial component. The capillaries tightly surround the basal membrane of the adenomatous structures. The identification of these endothelial cells of the capillary network is possible mostly by the immunohistochemistry for CD34. This intimate capillary network is not seen elsewhere [2, 6]. The stromal muscular component is made up of strands that grow among the epithelial component, resembling abortive vessels without the elastic layer. Occasionally myxoid, hyaline, or metaplastic change (ossification) is seen. This leiomyomyomatous stroma can also be seen in conventional clear cell renal cell carcinomas [2].

Leiomyomatous components in the tumor stroma of RAT are usually immunopositive for SMA, vimentin, and h-caldesmon while negative for HMB-45 and Melan-A. In the index case, the leiomyomatous component in the tumor stroma was positive for SMA. According to literature, few studies have reported renal cell tumors with glandular elements and leiomyomatosis stroma as a metachronous renal cell carcinoma with “an abnormally large quantity of smooth muscle, not related to the pelvis or calyces, nor to blood vessels” or describes them as “hamartomas” or “fibroleiomuscular” component. Kuhn et al. reported five cases of renal cell carcinomas with angioleiomyomas-like components and a desmoplastic reaction in the stroma, unlike what we see in RAT [1–3, 5].

MESTK is usually seen in middle-aged, peri-menopausal women and is related to estrogen. It was earlier grouped under the broader term of “Cystic nephroma.” The stroma in MESTK is identical to ovarian stroma with few leiomyomatosis areas. There can be various Müllerian epithelial type differentiations, e.g., tubal, endometrial, squamous. Intestinal mucinous glandular epithelium and Paneth cells may be seen. These features are not seen in RATs [6, 7]. Angiomyolipomas usually occur in association with tuberous sclerosis. They contain adipose tissue with thick blood vessels devoid of the elastic layer, which can sometimes be seen in RATs. However, they have a typical arrangement of myoid stromal cells which are perpendicular to vascular lumens. Also, angiomyolipomas, tumors stain positive for melanocytic markers like HMB45 and Melan-A, which is not seen in RATs. None of the melanocytic markers tested positive in the angioleiomyomatous stroma of RATs [7–9].

Conventional clear cell carcinomas may rarely show leiomyomatosis stroma. However, the characteristic Shark’s Smile is not seen. The VHL gene mutation and CD10 marker positivity are seen consistently in clear cell carcinomas and not found in RATs. Finally, we need to differentiate RATs from ccpRCC. Grossly both the tumor may show either cystic or papillary architecture. Rare cases of clear cell ccpRCC with RAT-like areas have been reported in the literature. In these cases, areas of ccpRCC will be evident with a minor component of the RAT-like area [10]. Immunohistochemical feature of RAT may overlap with ccpRCC, but morphologically ccpRCC will be having protuberant papillary architecture with thick cellular core and the large, generous clear cells lining the papillary structures so that the cells of one papilla may touch the cells of the adjacent papilla. In RAT, papillary structures are absent and the clear cell component is less prominent [6, 7, 10].

RAT usually a solid tumor with some microcystic areas. The presence of macro-cystic areas in RAT is a very rare occurrence. Michal et al. [2] studied five cases of RAT in his initial study out of which only one showed marked cystic areas. The present case tumor replaced the whole kidney and showed marked cystic changes which is an uncommon finding in RAT and not reported before. However, some studies like Deml et al. [11] have postulated that RAT and clear cell papillary renal cell carcinoma (ccpRCC) are two entities of the same spectrum of disease and it is difficult to distinguish on the grounds of morphology, immunohistochemistry markers, and molecular changes. They have described that RAT is a tumor with “varying amounts of tubular, papillary, and cystic architecture.” Other differentials include Xp11 and transcription factor E3 (TFE3) translocation cancer [8, 9, 11].

Precise diagnosis is crucial since this neoplasm has an excellent prognosis. Fluorescence in situ hybridization studies in four cases by Kuroda et al. have revealed that monosomy of chromosomes 1, 11, and 16 can be considered to be diagnostic in RAT. The preferred treatment is surgical resection and there have no reported cases of recurrence or death due to the neoplasm [10].

## Conclusion

To summarize, RAT is a rare renal neoplasm with variations in the presentation, gross, and microscopic findings, and having a benign course and good prognosis. However, owing to its distinct morphological, immunohistochemical, and genetic profile, a correct diagnosis needs to be made.

## Data Availability

The datasets used and/or analyzed during the current study are available from the corresponding author on reasonable request.

## Abbreviations

RAT: Renal angiomyoadenomatous tumor
WHO: World Health Organization
NCCT: Non-contrast computed tomography
PUJO: Pelvic ureteric junction obstruction
IHC: Immunohistochemistry
SMA: Smooth muscle actin
MESTK: Mixed epithelial and stromal tumor of the kidney
CcpRCC: Clear cell papillary renal cell carcinomas
PAS: Periodic acid-Schiff
